# Adaptation changes in dynamic postural control and contingent negative variation during repeated transient forward translation in the elderly

**DOI:** 10.1186/1880-6805-32-24

**Published:** 2013-12-19

**Authors:** Maki Maekawa, Katsuo Fujiwara, Naoe Kiyota, Chie Yaguchi

**Affiliations:** 1 Department of Human Movement and Health, Graduate School of Medical Science, Kanazawa University, 13-1 Takara-machi, Kanazawa 920-8640, Japan; 2 Department of Rehabilitation Science, Osaka Health Science University, 1-9-27 Tenma, Kita-ku, Osaka 530-0043, Japan; 3 Department of Physical Therapy, Faculty of Human Science, Hokkaido Bunkyo University, 5-196-1, Kogane-chuo, Eniwa 061-1449, Japan

**Keywords:** Adaptation, Anticipatory postural control, Contingent negative variation, Elderly subject, Floor translation, Postural disturbance, Postural muscle activation

## Abstract

**Background:**

Adaptation changes in postural control and contingent negative variation (CNV) for the elderly were investigated during repeated forward floor translation.

**Methods:**

Fifteen healthy elderly persons, living in the suburban area of Kanazawa City, Japan, underwent backward postural disturbance by a forward-floor translation (S2) 2 s after an auditory warning signal (S1). A set with 20 trials was repeated until a negative peak of late CNV was recognized in the 600-ms period before S2, and the last set was defined as the final set. Electroencephalograms, center of foot pressure in the anteroposterior direction (CoPap), and electromyograms of postural muscles were analyzed.

**Results:**

CoPap displacement generated by the floor translation was significantly decreased until the twelfth trial in the first set, and mean CoPap displacement was smaller in the second and final sets than in the first set. The mean displacement was significantly smaller in the final set than the previous set. A late CNV with a negative peak was not recognized in the first and second sets. However, most subjects (13/15) showed a negative peak by the fourth set, when the late CNV started to increase negatively from about 1,000 ms after S1 and peaked at about 300 ms before S2. At about 160 ms before the CNV peak, the CoPap forward shift started. The increase in timing of the gastrocnemius activity related to the CoPap shift was significantly correlated with the CNV peak timing (*r* = 0.64). After S2, peak amplitudes of the anterior postural muscles were significantly decreased in the final set compared to the first set.

**Conclusions:**

It was demonstrated that even for the elderly, with so many repetitions of postural disturbance, a late CNV with a negative peak was recognized, leading to accurate postural preparation. This suggests the improvement of frontal lobe function (e.g., anticipatory attention and motor preparation) in the elderly.

## Background

Deterioration of equilibrium function is considered to be a primary cause of falls in the elderly. Falls often occur in a dynamic situation, such as when perturbation is externally applied and during walking. Backward falls pose a greater risk for serious hip and head injuries due to the absence of backward visual information [1,2]. Thus, it is meaningful to develop equilibrium training to prevent backward falls in the elderly. For training, forward floor translation can be applied to cause backward disturbance, as this manipulation resembles slips during walking [3-5].

In order to adapt to a postural disturbance, the anticipation of disturbance timing and preparation for the disturbance are required functions [6,7]. The main brain parts executing these functions are the frontal lobe, including the prefrontal cortex, supplementary motor area, premotor area, and primary motor area [8,9]. Although studies of postural control have reported more marked training effects even in the elderly [10,11], there have been few experimental studies using electroencephalography (EEG) to directly address training effects on the frontal lobe function related to dynamic postural control in the elderly [12].

Postural control functions of the frontal lobe have been evaluated with measurement of the contingent negative variation (CNV) of EEG in a S1 (warning signal) to S2 (response signal) paradigm [13]. It has been suggested that the late component of CNV (late CNV) is related to the activation of various cortical and subcortical regions, such as frontal cortical areas, the somatosensory cortex, and basal ganglia [14]. Late CNV is reported to reflect the motor preparation process and anticipatory attention directed to S2 [15,16], and its amplitude increases with larger amounts of attention directed to S2 [16]. It has been suggested that a negative peak of the late CNV corresponds to a critical point of anticipatory attention and/or onset of attentional shift to objects other than S2, such as sensory information and output of motor commands [17]. In our previous studies, floor translation has been used as S2 [12,18,19]. Across 40 postural disturbances using forward floor translation, we investigated adaptation changes in dynamic postural control and CNV [12]. In the process of the adaptation, the late CNV negatively increased in later trials for young adults, but the CNV with the negative peak was not found in the elderly. For the young adults, corresponding to the CNV peak before disturbance, the forward shift of center of foot pressure in the anteroposterior direction (CoPap) and/or the increase of postural muscle activities started just before S2.

In previous CNV studies with finger reaction tasks for the elderly, the negative peaks of late CNV were reported to be small or absent [14,20]. This finding would suggest age-related deterioration of anticipatory attention and/or motor preparation. However, in the previous study related to the adaptability with finger movement tasks, the elderly were able to perform the complex task as automatically as young adults, although more trials were needed. Further, in the adaptation stage, the activated brain area during the task was larger in the elderly than in the young [21]. On the other hand, Kiyota and Fujiwara have reported that after anti-saccade training for 3 weeks, performance improved even for the elderly, and the peak amplitude of pre-saccadic brain potential increased, which is considered as an index of information processing and activation in the frontal brain regions [22]. These results suggest that even for the elderly, the frontal lobe function related to those tasks could be improved with repetitive training.

Therefore, for the elderly, forward floor translation was applied more than 40 times, and adaptation changes in postural control and late CNV were investigated. The working hypotheses were as follows: i) postural stability would be improved in the early stage, and with so many repetitions of the disturbance, CNV potential would increase negatively toward S2 and show the negative peak just before S2; ii) corresponding to the CNV peak before the disturbance, the forward shift of CoPap and/or the increase of postural muscle activities would start just before S2.

## Methods

### Subjects

Fifteen healthy elderly adults (7 men, 8 women) participated in this experiment. Mean ± standard deviation (SD) of age, height, weight, foot length (FL), and auditory threshold were 71.2 ± 6.0 years, 156.7 ± 10.0 cm, 58.4 ± 9.1 kg, 23.6 ± 1.6 cm, and 41.9 ± 8.0 dB, respectively. The subjects lived in the suburban area of Kanazawa City, Japan, and could perform activities of daily living without assistance. No subject had any history of neurological or orthopedic impairment. Informed consent was obtained from all subjects in accordance with the Declaration of Helsinki following an explanation of our experimental protocols, which were approved by the Institutional Ethics Committee of Kanazawa University.

### Apparatus and data recording

A platform (FPA34; Electro-design, Japan) was used to measure CoPap. The CoPap electronic signals were sent simultaneously to two computers: one of them (PC9801BX2; NEC, Japan) to determine CoPap position and the other (Dimension E521; Dell Japan, Japan) for analysis. The former received CoPap data via an A/D converter (PIO9045; I/O-Data, Japan) at 20 Hz with 12-bit resolution, and generated a buzzer sound when CoPap was located within ±1 cm of the required position. CoPap position was calculated and represented as the percentage distance from the heel in relation to FL (%FL). The force platform was fixed to a handmade table that was movable horizontally in an anteroposterior direction by a computer-controlled electric motor (VLA-ST-60-60-0300; THK, Japan). Direction, velocity, and amplitude of platform movement were adjusted using Cutey Wave II software (Sanmei-Denshi, Japan). S1 was an auditory stimulus delivered via earphones with frequency, intensity, and duration of 2,000 Hz, 35 dB above the threshold, and 50 ms, respectively. The onset of a transient forward floor translation was used as S2, which was detected by an accelerometer (AG-2GB; Kyowa, Japan) fixed to the force platform. To measure the platform movement, a position sensor system (C5949; Hamamatsu Photonics, Japan) was used.

Ag-AgCl cup electrodes (diameter, 8 mm) for recording EEG were affixed to the scalp at Fz, Cz, and Pz in accordance with the international 10–20 system, and referenced to linked ear lobes; a ground electrode was placed at Fpz. Electrooculography (EOG) was recorded from a pair of electrodes placed above and below the left eye. To fix eye position, subjects were instructed to gaze at a fixation point presented on an Eye-trek face-mounted display (FMD011F; Olympus, Japan). Surface electrodes (P-00-S; Ambu, Denmark) were used in bipolar derivation to record surface electromyography (EMG) of the following muscles on the left side: rectus abdominis (RA); erector spinae; rectus femoris (RF); biceps femoris; tibialis anterior (TA); medial head of gastrocnemius (GcM); and soleus. For each muscle, electrodes were fixed after shaving and cleaning the skin with alcohol. The electrodes were aligned along the long axis of the muscle with an inter-electrode distance of about 3 cm. Electrode input impedance was <5 kΩ. Signals from electrodes were amplified (EEG, ×40,000; EOG, ×4,000; EMG, ×4,000) and band-pass filtered (EEG, 0.05–100 Hz; EOG, 0.05–30 Hz; EMG, 5–500 Hz) using an amplifier (Biotop 6R12; NEC-Sanei, Japan).

For subsequent analyses, all electrical signals were simultaneously sent to the computer for analysis via an A/D converter (ADA16-32/2(CB) F; Contec, Japan) at 1 kHz with 16-bit resolution.

### Procedure

**Figure 1:**
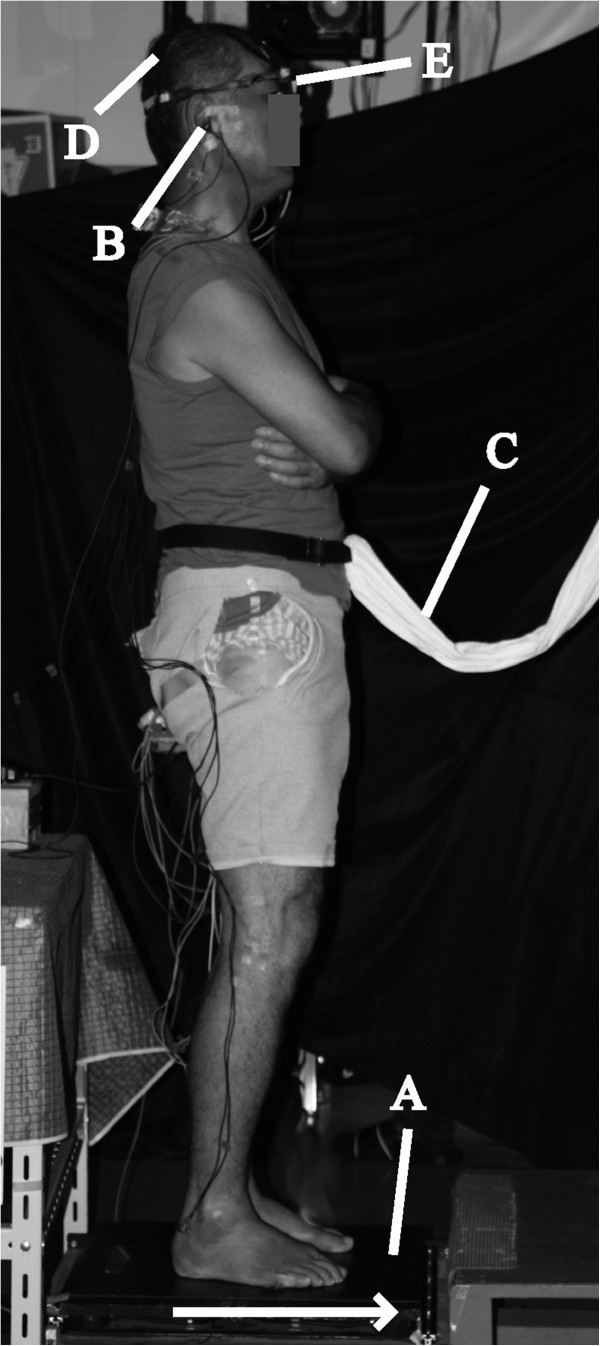
**Experimental setup. (A)** Platform; **(B)** Earphone; **(C)** Harness; **(D)** Ag-AgCl cup electrodes; **(E)** Eye-trek face-mounted display. Direction of platform movement is indicated by an arrow.

All measurements were carried out while subjects were standing barefoot, with feet 10 cm apart and parallel on the force platform, and upper limbs crossed in front of the chest. To prevent falls due to floor translation, subjects wore a harness around the waist (Figure 1).

First, the mean positions of CoPap were measured while subjects maintained a quiet standing (QS) posture for 10 s. The mean value of the five trials was adopted as the QS position. Next, mean CoPap position during extreme backward leaning (EBL) posture was measured twice. Subjects gradually leaned backward from QS for approximately 5 s, pivoting at the ankles with the rest of the body kept aligned, and then maintained this EBL posture for 3 s. The more posterior CoPap mean position of the two trials was adopted as the EBL mean position, and the posterior peak position of CoPap in the adopted trial was defined as the EBL peak position.

Preceding the experimental session, in order to apply a relatively equal intensity of postural disturbance among the subjects, the velocity and amplitude of floor translation was set for each subject, based on the EBL mean and peak positions, as follows [23]. First, the translation velocity was determined. To begin, 10 cm/s floor translation was applied at 5- and 10-cm amplitudes. If the posterior peak of CoPap after the translation at either amplitude was located between EBL mean and peak positions, 10 cm/s was adopted as the translation velocity. If not, the velocity was reduced or increased until the posterior peak at either amplitude was located between these positions. Second, a linear regression line was drawn through the two coordinates of the floor translation amplitude and the posterior peak of CoPap at the determined velocity. Based on the line, the translation amplitude, at which the posterior peak would be located midway between EBL mean and peak positions, was determined. The mean ± SD of the adopted translation velocity and amplitude were 14.9 ± 4.2 cm/s and 7.6 ± 2.0 cm, respectively.

The experimental session was carried out as follows. In both the setting of the translation intensity and the experimental session, subjects maintained CoPap position within the QS position ±1 cm, which was presented by a buzzer sound for at least 3 s, until S2 onset. S1 was randomly presented 1 to 2 s after the experimenter stopped the buzzer sound, and then S2 started 2 s after S1. Subjects were instructed to avoid changing their initial foot position in response to S2. A set with 20 trials was repeatedly performed (2–4 sets per subject (mean was 3)) until the late CNV with a negative peak (CNV peak) was recognized between 1,400 and 2,000 ms after S1, which is the range in which the CNV peak appeared in the previous young subjects [12]. Trials were excluded if foot position was changed, or if CoPap deviated over ±1 cm from the QS position before S2. Subjects had a standing rest period of 30 s between trials and a seated rest period of 3 min and 5 min between every 10 trials and between each set, respectively.

### Data analysis

All data analysis was performed using BIMUTAS II software (Kissei Comtec, Japan). To evaluate the magnitude of backward disturbance in response to the floor translation, the posterior peak of CoPap after S2 was identified in each trial and CoPap displacement was evaluated by the distance from EBL mean position to this peak position. Moreover, mean CoPap displacement of all trials in each set was calculated to investigate the relationship with CNV.

EEG, EMG, and CoPap waveforms from 500 ms before S1 to 3,000 ms after S2 were averaged for each set. Trials with eye blinks or movement artifacts (voltage at EOG or any EEG electrode exceeding ±100 μV) between 500 ms before S1 and S2 were excluded from the averaging, and at least 12 trials were adopted for each set. The mean amplitude for the 500-ms period before S1 was used as a baseline of averaging. Preceding EMG averaging, all EMGs were 40-Hz high-pass-filtered to exclude electrocardiographic and movement artifacts and then full-wave-rectified (rEMG). Averaged waveforms of EEG, rEMG, and CoPap between S1 and S2 were 4-Hz low-pass filtered.

Waveforms recorded from Cz, in which late CNV was maximum in all sets, were used for CNV analyses. In each averaged waveform, mean amplitudes for every 100-ms period between S1 and S2 were calculated. CNV can be classified into early and late components [24-26]. It has been reported that the early component is the potential between 300 ms and 700 ms after S1 [27-29], and the late component is the negative potential, which gradually increases toward S2 [13,26,27]. We confirmed that the peak of early component appeared 700 ms after S1 (the mean latency was 514.1 ± 88.2 ms); thus, mean amplitudes in the periods from 700 ms after S1 to S2 were used for the analysis of late CNV. A maximal negative potential identified from 1,400 ms after S1 to S2 was defined as the CNV peak, and its latency relative to S2 was calculated as CNV peak time. The set with the CNV peak was defined as the final set.

In the previous studies using a S1-S2 paradigm with floor translation as S2, it has been reported that the continuous increase of EMG background activity and CoPap shift toward S2 started around the CNV peak point and high correlations were found between CNV peak time and onset times of the EMG increase and CoPap movement [12,18]. Therefore, EMG increase and CoPap shift before S2 were determined in the final set, using the averaged waveforms. Increasing timing of GcM was identified as the point in which the amplitude first exceeds the mean amplitude of S1-S2 period for >50 ms from around CNV peak to S2 and the start time relative to S2 was calculated [12]. The start time of the CoPap shift was also calculated in the same way.

For the first burst activity of anterior muscles after S2 (RA, RF, and TA), the following analyses were conducted in the trials adopted for averaging. In each trial, the envelope line of the EMG burst, continuing at least 50 ms, was identified by visual inspection of the EMG trace on a computer. The burst onset was defined as the point at which the EMG deviated more than the mean +2 SD of the background activity during the standing posture before S1, and the onset time from S2 was measured. Then, rEMG waveforms from −500 ms to +1,000 ms with respect to the burst onset were averaged for each set. The mean amplitude of the 300-ms period before S1 was used as a baseline for averaging. The average waveforms were smoothed using a 40-Hz low-pass filter, and then a peak was identified [30]. The peak amplitude from the baseline and peak time with respect to burst onset were measured. The integrated EMG from the burst onset point to the peak point was calculated.

### Statistical analysis

Because 2 of 15 subjects complained of severe fatigue during the fourth set, they could not accomplish the experiment and their CNV peaks could not be obtained. Therefore, the comparisons between the first and second sets were conducted for all subjects, and comparisons between the first and final sets were conducted for the 13 subjects with CNV peaks.

Shapiro-Wilks and Levene’s tests confirmed that all data satisfied the assumptions of normality and equal variance, respectively. To compare EBL mean position and CoPap displacement in this study with those in the previous study [12], the Student’s *t*-test was used. To evaluate set differences in CoPap displacement and EMG parameters after S2, the paired *t*-test was used. One-way repeated-measures analysis of variance (ANOVA) was used to assess the changes in CoPap displacement with repetition of trials. When a significant main effect of the trial was shown, the differences between the first trial and the subsequent trials were evaluated by Dunnett’s one-to-many post-hoc test. A two-way ANOVA was used to assess the effects of set and period (2 sets × 13 periods) on the CNV mean amplitude for every 100-ms period between 700 ms after S1 and S2. When significant main effects of set and period were shown, and significant interaction between the two factors was shown, the difference between the period of 700–800 ms after S1 and the other periods were evaluated using the Dunnett’s test by set, and the difference between sets in each period were evaluated using the paired *t*-test. Pearson’s correlation was used to assess the relationship between CNV peak time and both onset time of EMG increase and CoPap shift before S2. The alpha level for all tests was set at *P* <0.05. All statistical analyses were performed using SPSS 14.0 J software (SPSS Japan).

## Results

Mean ± SD of the QS position, and EBL mean and peak positions were 43.3 ± 5.5%FL, 20.4 ± 1.6%FL, and 14.6 ± 1.7%FL, respectively. The EBL mean position was not significantly different to the previous data on elderly subjects (21.8%FL) [12], but was more forward than the previous data on young subjects (18.6%FL) [12] (*t*_20_ = 2.33, *P* <0.05).

**Figure 2:**
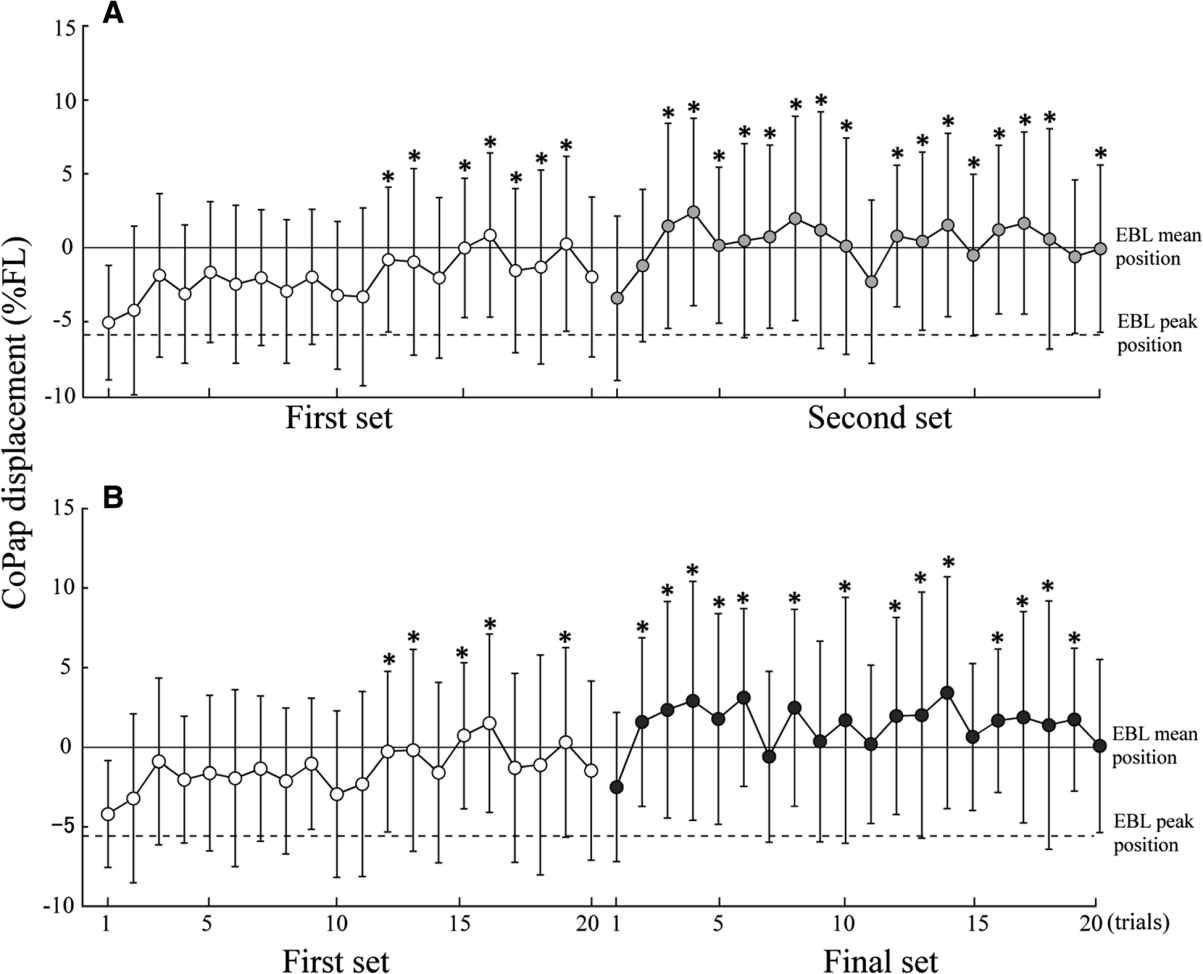
**Means and standard deviations of CoPap displacement in response to the floor translation in each trial. (A)** Comparison between the first and second sets for 15 subjects; **(B)** Comparison between the first and final sets for 13 subjects. The asterisk indicates a significant difference on a trial compared to the first trial in the first set (*: *P* <0.05).

**Figure 3:**
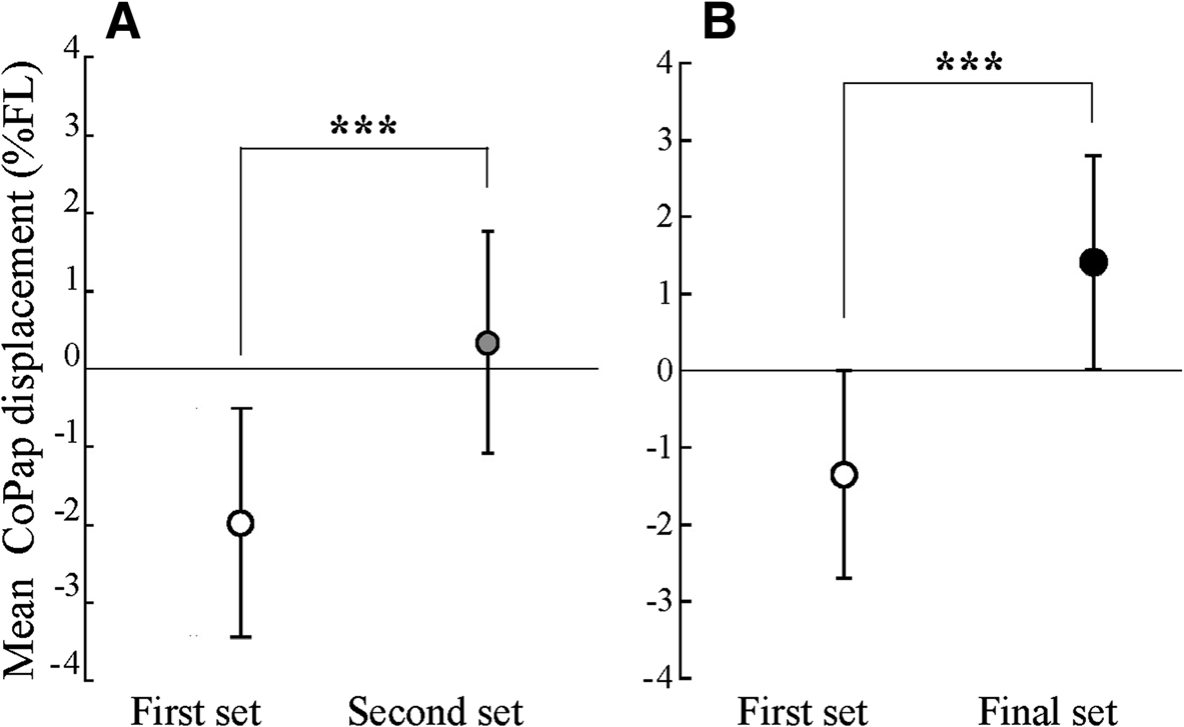
**Means and standard deviations of mean CoPap displacement among 20 trials in each set. (A)** Comparison between the first and second sets for 15 subjects; **(B)** Comparison between the first and final sets for 13 subjects. The asterisk indicates a significant difference between sets (***: *P* <0.001).

Figure 2 shows CoPap displacement in response to the FL in each trial. The CoPap displacement in the first trial of the first set was −5.1 ± 3.9%FL and was not significantly different with the previous data on elderly subjects (−3.8%FL) [12]. Compared with the first trial of the first set, the CoPap displacement was significantly decreased after the 12^th^ trial of the first set (*P* <0.05) for both the 15 subjects (Figure 2A, the first set: *F*_19, 266_ = 2.76, *P <*0.001; the second set: *F*_20, 280_ = 2.86, *P <*0.001) and the subset of 13 subjects (Figure 2B, the first set: *F*_19, 209_ = 2.38, *P <*0.01; the final set *F*_20, 240_ = 1.68, *P <*0.05). Mean CoPap displacement in the second set (*t*_14_ = 4.16, *P <*0.001) was significantly smaller than the first set (Figure 3A). The mean CoPap displacement in the final set was significantly smaller than that in the first set (Figure 3B, *t*_12_ = 3.55, *P <*0.01) and in the set just before the final set (*t*_7_ = 2.93, *P <*0.05). From the four subjects who participated in the fourth experimental set, there was no significant difference in the mean CoPap displacement between the second and third sets.

**Figure 4:**
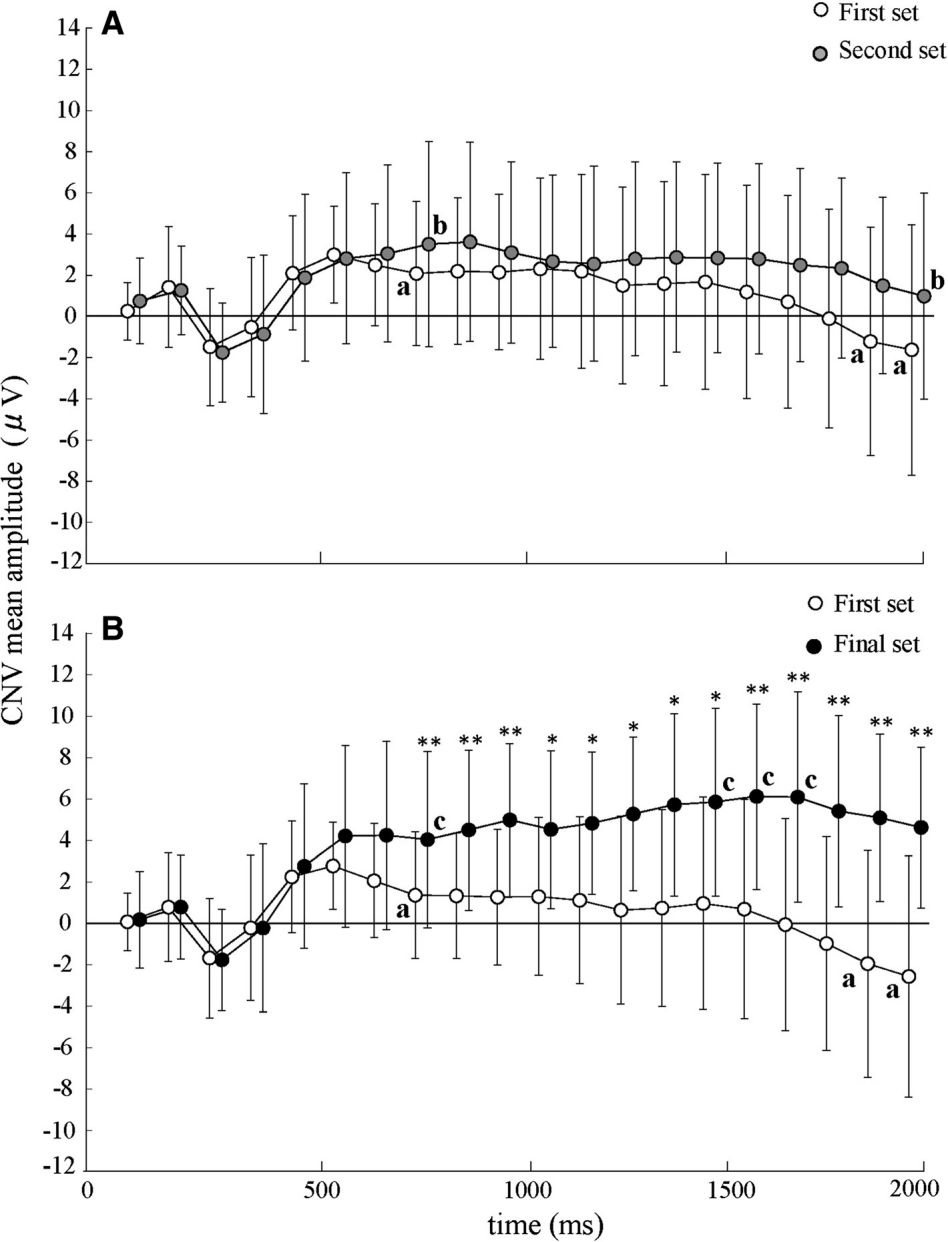
**Means and standard deviations of mean amplitudes for every 100-ms period of CNV between S1 and S2. (A)** Comparison between the first (white circle) and second (gray circle) sets for 15 subjects; **(B)** Comparison between the first (white circle) and final (black circle) sets for 13 subjects. The asterisk indicates a significant increase on a trial in the final set compared with the first set (*: *P* <0.05, **: *P* <0.01). ‘a’ and ‘b’ indicate a significant decrease from the 700–800 ms period after S1 and ‘c’ indicates a significant increase from that, respectively.

Means and SD of mean amplitude for every 100-ms period of CNV between S1 and S2 are shown in Figure 4. CNV peak was not found for any subjects in the first set, but was found in 5 in the second set, 4 in the third set, and 4 in the fourth set. When the CNV mean amplitude was compared between the first and second sets, a significant effect of period (*F*_2, 27_ = 5.03, *P <*0.05) was found, but there was no significant effect of sets or interaction between periods and sets (Figure 4A). Compared with the period of 700–800 ms after S1, CNV mean amplitudes decreased in the periods from 1,800 ms to 2,000 ms in the first set, and in the period from 1,900 ms to 2,000 ms in the second set (*P <*0.05). In the comparison between the first and final set, there were significant effects of sets (*F*_1, 12_ = 13.28, *P <*0.01) and periods (*F*_3, 32_ = 2.87, *P <*0.05) and interactions between them (*F*_3, 35_ = 4.42, *P <*0.01) (Figure 4B). Compared with the period of 700–800 ms after S1, the CNV mean amplitude decreased in the periods from 1,800 ms to 2,000 ms in the first set, while it increased in the periods from 1,400 ms to 1,700 ms in the final set (*P <*0.05). The CNV mean amplitude in every period was significantly larger in the final set than in the first set (*P <*0.05).

In the final set, GcM and CoPap started to increase and shift forward, respectively, from around the CNV peak to S2 (N = 9). Three subjects showed bilateral activation of the lower leg muscles (TA and GcM). In one subject, there was a transient increase of TA activation just before S2 without the CoPap shift. The onset time of GcM increase and CoPap forward shift were 511.3 ± 132.4 ms and 494.7 ± 150.6 ms, respectively. Moreover, these onset times significantly preceded the CNV peak (GcM increase and CoPap shift: 178.7 ± 138.6 ms and 162.0 ± 114.8 ms, respectively) (*t* >4.46, *P <*0.001). Further, the onset time of the GcM increase and CoPap forward shift showed significant correlations with the CNV peak time (*r* = 0.64, *P <*0.05 and *r* = 0.77, *P <*0.01, respectively).

For the EMG activation after floor translation, the burst onset time, peak time, peak amplitude, and integrated EMG are shown in Table 1. The peak amplitude of RA, RF, and TA were significantly decreased in the final set compared to the first set (RA: *t*_*12*_ = 2.09, *P <*0.05; RF: *t*_*12*_ = 2.94, *P <*0.05; TA: *t*_*12*_ = 2.90, *P <*0.05). The integrated EMG of the RF and TA were significantly smaller in the final set than the first set (RF: *t*_*12*_ = 2.88, *P <*0.05; TA: *t*_*12*_ = 2.19, *P <*0.05). In the burst onset time and peak time, there were no significant differences between the first and final sets. No significant differences in any measurement were found between the first and second sets.

**Table 1:** Means and standard deviations for the EMG activation after floor translation

		**RA**	**RF**	**TA**
Burst onset time	First set	175.8 ± 16.9	121.2 ± 19.5	106.3 ±10.7
(ms)	Final set	172.8 ± 25.9	117.4 ±20.5	104.2 ±11.4
Peak time	First set	590.1 ± 84.5	599.5 ± 45.2	582.4 ± 63.4
(ms)	Final set	550.8 ± 36.1	581.6 ±33.6	543.8 ± 38.0
Peak amplitude	First set	5.4 ± 3.8*	85.8 ± 47.1*	295.0 ± 113.4*
(μV)	Final set	4.6 ± 3.2*	73.2 ± 46.0*	269.0 ± 109.0*
Integrated EMG	First set	242.1 ± 178.7	5403.4 ± 3638.2*	13379.3 ± 8667.8*
(μV ms)	Final set	177.7 ± 156.7	4063.8 ± 2658.1*	7447.3 ± 3634.3*

Asterisk indicates a significant difference between sets (*: *P* <0.05).

## Discussion

EBL mean position is an index of the posterior limit of stability [31]. No significant differences in either the EBL mean position or CoPap displacement were found between the elderly subjects in the previous study [12] and those in the present study. These results suggest that the intensity of floor translation could be set similarly between the previous and present subjects.

EBL mean position was more forward in the present elderly subjects than in the previous young subjects (18.6% FL) [12]. In addition, CoPap displacement significantly decreased after the 12^th^ trial in the first set. The number of trials for the adaptation was lower compared with the previous elderly subjects (19^th^ trial) [12]. However, within the trials after the 12^th^ trial, there were less trials with a significant difference compared to the first trial for the elderly subjects of the present study than for the young subjects in the previous study. Mean CoPap displacement in the second set was significantly smaller than the first set. These results indicated that even for the elderly, anticipatory postural control was clearly improved by the second set, but the improvement occurred slightly later compared with the young subjects. For the improvement of postural stability in the early stage, the cerebellum, which has a main role in automatic postural control, closely contributes [32,33].

As in the previous study [12], late CNV with a negative peak was not recognized in the first and second sets. However, with more repetitions of postural disturbance, late CNV was found by the fourth set in most subjects (13 of 15 subjects). It has been considered that late CNV is related to the activation of various cortical and subcortical regions, such as frontal cortical areas, the somatosensory cortex, and basal ganglia [14], and reflects the motor preparation process and anticipatory attention directed to S2 [15,16]. Using various measures for the elderly, negative CNV amplitude was reported to be small or absent [14,20]. This finding would suggest age-related deterioration of anticipatory attention and/or motor preparation. On the other hand, also in the button press task under anxious or fearful conditions, late CNV with a negative peak was not found [34,35]. For the present subjects, in the sets in which the late CNV was not recognized, postural controllability was inferior to the young subjects. Therefore, in the first and second sets, and due to their lower postural controllability and their anxiety or fear of postural disturbance, the subjects may direct their attention to the other object of S2 from the early phase.

However, even for the elderly, in the final set after so many repetitions of postural disturbance, late CNV with a negative peak was found. It is possible that the subjects could direct lots of anticipatory attention to the postural disturbance and sufficiently prepare for it. This focusing of attention would be related to the improvement of postural stability and the relief of their anxiety or fear to the disturbance. Such adaptational change in the late CNV occurred following that in CoPap displacement. Moreover, mean CoPap displacement in the final set was significantly smaller than in the preceding set. This suggests that sufficient attention and motor preparation to S2 might cause further improvement of the postural stability, mainly relating to the frontal lobe function.

In the final set, many subjects showed a forward shift of CoPap and an increase of GcM background activity from around the CNV peak point to the floor translation onset, which would represent postural preparatory adjustment against the disturbance. The onset timing of the EMG increase was 178.7 ± 138.6 ms before the CNV peak. This delay of the CNV peak from the EMG increase would reflect the attention directed to the processes of sensory information from postural muscles [12,36]. It is considered that when the late CNV with a negative peak is recognized, sufficient postural preparation could be executed, based on the accurate prediction of the disturbance timing. Also in the final set, EMG activation was significantly decreased just after the floor translation, in spite of no changes in the timing of the EMG activation onset and the time until the EMG peak. This suggests that sufficient postural preparation against the disturbance would lead to effective postural control.

## Conclusions

It was demonstrated herein, that following so many repetitions of postural disturbance, the late CNV with a negative peak was recognized even for the elderly, leading to accurate postural preparation and effective postural control. This suggests that improvement of frontal lobe function (e.g., anticipatory attention and motor preparation) can occur in the elderly following sufficient repetition.

## Abbreviations

CNV: Contingent negative variation; CoPap: Center of pressure in the anteroposterior direction; EBL: Extreme backward leaning; EEG: Electroencephalography; EMG: Electromyography; EOG: Electrooculography; FL: Foot length; GcM: Gastrocnemius; %FL: Percentage distance from the heel in relation to FL; QS: Quiet standing; RA: Rectus abdominis; rEMG: Full-wave-rectified EMG; RF: Rectus femoris; S1: Warning signal; S2: Response signal; TA: Tibialis anterior.

## Competing interests

The authors declare that they have no competing interests.

## Authors’ contributions

Contribution of each author is as follows: MM and KF presented all the idea of this study, planed the method, directed the experiments and interpreted the results. Most of the article was written by MM and KF. NK and CY contributed to the experiments, data analyses, and in manuscript preparation. NK and CY discussed the manuscript with MM and KF. All authors have approved the revised article.
